# Protocol: An improved high-throughput method for generating tissue samples in 96-well format for plant genotyping (Ice-Cap 2.0)

**DOI:** 10.1186/1746-4811-3-8

**Published:** 2007-06-12

**Authors:** Katie A Clark, Patrick J Krysan

**Affiliations:** 1 Genome Center of Wisconsin and Department of Horticulture, 1575 Linden Drive, University of Wisconsin-Madison, Madison, WI 53706 USA

## Abstract

**Background:**

We previously developed a high-throughput system called 'Ice-Cap' for growing *Arabidopsis *seedlings in a 96-well format and rapidly collecting tissue for subsequent DNA extraction and genotyping. While the originally described Ice-Cap method is an effective tool for high-throughput genotyping, one shortcoming of the first version of Ice-Cap is that optimal seedling growth is highly dependent on specific environmental conditions. Here we describe several technical improvements to the Ice-Cap method that make it much more robust and provide a detailed protocol for implementing the method.

**Results:**

The key innovation underlying Ice-Cap 2.0 is the development of a continuous watering system. The addition of the watering system allows the seedling growth plates to be incubated without a lid for the duration of the growth period, which in turn allows for much more uniform and robust seedling growth than was observed using the original method. We also determined that inserting wooden skewers between the upper and lower plates prior to tissue harvest made it easier to separate the plates following freezing. Seedlings grown using the Ice-Cap 2.0 method remain viable in the Ice-Cap plates twice as long as seedlings grown using the original method.

**Conclusion:**

The continuous watering system that we have developed provides an effective solution to the problem of sub-optimal seedling growth that can be encountered when using the originally described Ice-Cap system. This novel watering system and several additional modifications to the Ice-Cap procedure have improved the robustness and utility of the method.

## Introduction

Plant scientists increasingly need to genotype large numbers of individual plants as a part of their research programmes. For example, when performing map-based cloning to locate the genetic lesion responsible for a specific phenotype, it is often necessary to genotype thousands of individual plants from segregating populations. For crop species, the process of marker-assisted selection can involve genotyping several thousand individuals in a large population. To optimize the efficiency with which these types of experiments can be performed, it is necessary to develop a streamlined plant genotyping pipeline. We previously described a method named "Ice-Cap" to address these needs [1]. Ice-Cap is a novel high-throughput system for growing plant seedlings and collecting tissue in 96-well plate format. The key innovation in Ice-Cap is that tissue harvest for all 96 seedlings is performed in parallel, so that single plant manipulations are not necessary. This feature of Ice-Cap sets it apart from other high-throughput genotyping methods that rely on collecting leaf tissue samples one-at-a-time from individual plants, which constitute a major bottleneck in a genotyping pipeline [2-4]. While the original version of Ice-Cap is a useful tool, we have made several significant improvements that enhance the performance of the system and allow for more robust seedling growth. These improvements are described here, along with a detailed protocol.

## Materials

### Consumables

Murashige and Skoog (MS) basal salt mixture e.g. Sigma-Aldrich, St. Louis, (catalogue no. M5524).

4-Morpholinoethanesulfonic acid (MES) e.g. Sigma-Aldrich, (catalogue no. M-2933).

Plant cell culture tested agar e.g. Sigma-Aldrich (catalogue no. A9915-1KG).

Sterile pipette tips, 20–200 μL size.

Multi-channel pipette trough e.g. Fisher Scientific 50 ml pipette basin (Fisher catalogue no. 13-681-500).

Surgical tape e.g. 3 M Micropore surgical tape (Fisher catalogue no. 19-027-761).

96-well deep well plates (referred to as 'upper seedling plates' in the Ice-Cap protocol) e.g. Fisher Scientific Nunc brand, 1-mL filter plates without frit (Fisher catalogue no. 278012). These plates are manufactured with a hole in the bottom of each well to allow roots to grow down into lower root plates.

96-well PCR plates (referred to as 'lower root plates' in the Ice-Cap protocol) e.g. Fisher thin-wall PCR plates with raised rims (Fisher catalogue no. 0550127). These PCR plates have a raised rim to fit securely onto the upper seedling plates.

Clear plastic lids e.g. lids taken from Falcon Microtest flat bottom 96-well polystyrene plates (Falcon catalogue no. 351172). Lids must be correct size to fit onto tops of upper seedling plates.

Metal ball bearings e.g. 3/32-inch diameter stainless steel balls (Hartford Technologies, Rocky Hill, CT; catalogue no. 034-006-1K).

Adhesive sealing film e.g. Fisher Scientific (Fisher catalogue no. 05-500-32).

Elastic bands e.g. 7.5 cm (3") elastic hair bands purchased from grocery store.

4 mm diameter wooden skewers. Purchased from a grocery store. These skewers are the type that is typically used to prepare kabobs.

Dry ice.

95% (v/v) ethanol

Thermal adhesion foil e.g. Easy Peel heat sealing foil (catalogue no. AB-0745, ABgene USA, Rochester, NY)

### Equipment

A pH meter

Multi-channel pipettors with volume ranges from 20 μL to 180 μL

A 96-well *Arabidopsis *seed-loading device (V&P Scientific, San Diego, CA)

Large, shallow metal pan with a rim e.g. 43.8 cm × 30.8 cm × 2.5 cm (17.25" × 12.15" × 1") cookie sheet. We use a rimmed metal cookie sheet (Chicago Metallic, Vernon Hills, IL), available from a cooking supply store. The pan should be just deep enough so that when the pan is filled with water the water will just cover the tops of wells of the lower root plate without submerging upper seedling plate. To achieve the desired water depth it may be necessary to place a thin sheet of glass on the bottom of the cookie sheet.

A small submersible water pump e.g. small tabletop fountain pump (PT-295, Rolf C. Hagen Corp., Mansfield, MA), available from a garden supply store.

A 26-L polypropylene storage box e.g. 53.3 cm × 38.1 cm × 14 cm (21" × 15" × 5.5") (Sterilite Co., Townsend, MA) or other similar watertight container. Container must be longer and wider than the metal pan to allow water to overflow from the pan down into the container. Container must be deep enough to completely submerge fountain pump.

A metal rack. We placed the metal pan on a custom-made rack constructed of 2.5 cm (1") metal bars with adjustable screws for levelling the metal pan. This metal rack was held above the bottom of the 26-L storage box by the plastic ledges present in the sides of the storage box.

Some 12.7 mm (1/2") diameter plastic tubing or size appropriate for the fountain pump.

Small water-resistant clamps which are used to secure the plastic tubing to the side of the metal pan.

A hand-held microseal roller e.g. Roller for Microseal Film MJ Research, Inc. (Waltham, MA, catalogue no. MSR-0001).

A 1–2 cm wide soft bristle artist's paintbrush, purchased from a craft store.

A manual heat-sealer e.g. Thermo-Sealer from ABgene USA (Rochester, NY, catalogue no. AB-0384).

A 96-well metal thermal block e.g. Cole-Parmer 96-well microplate heating block module (catalogue no. 36400-66).

A glass dish e.g. Pyrex 2-L baking dish, 11 cm × 7.5 cm × 4 cm (11" × 7.5" × 1.75"), purchased at a cooking supply store. Dish is used for dry ice/ethanol bath and must be freezing tolerant.

A Genogrinder or paint shaker modified to hold 96-well plates e.g. 2000 Geno/Grinder machine (Spex/CertiPrep, Metuchen, NJ).

A centrifuge equipped with microplate carriers e.g. Beckman Coulter Allegra™ 25R Centrifuge equipped with S5700 rotor and microplate swing buckets.

Some 1 cm diameter rubber tubing or appropriate size to attach to compressed air supply for use in recovering individual seedlings from Ice-Cap plates after genotyping has been completed.

### Continuous Watering System

**Figure 1:**
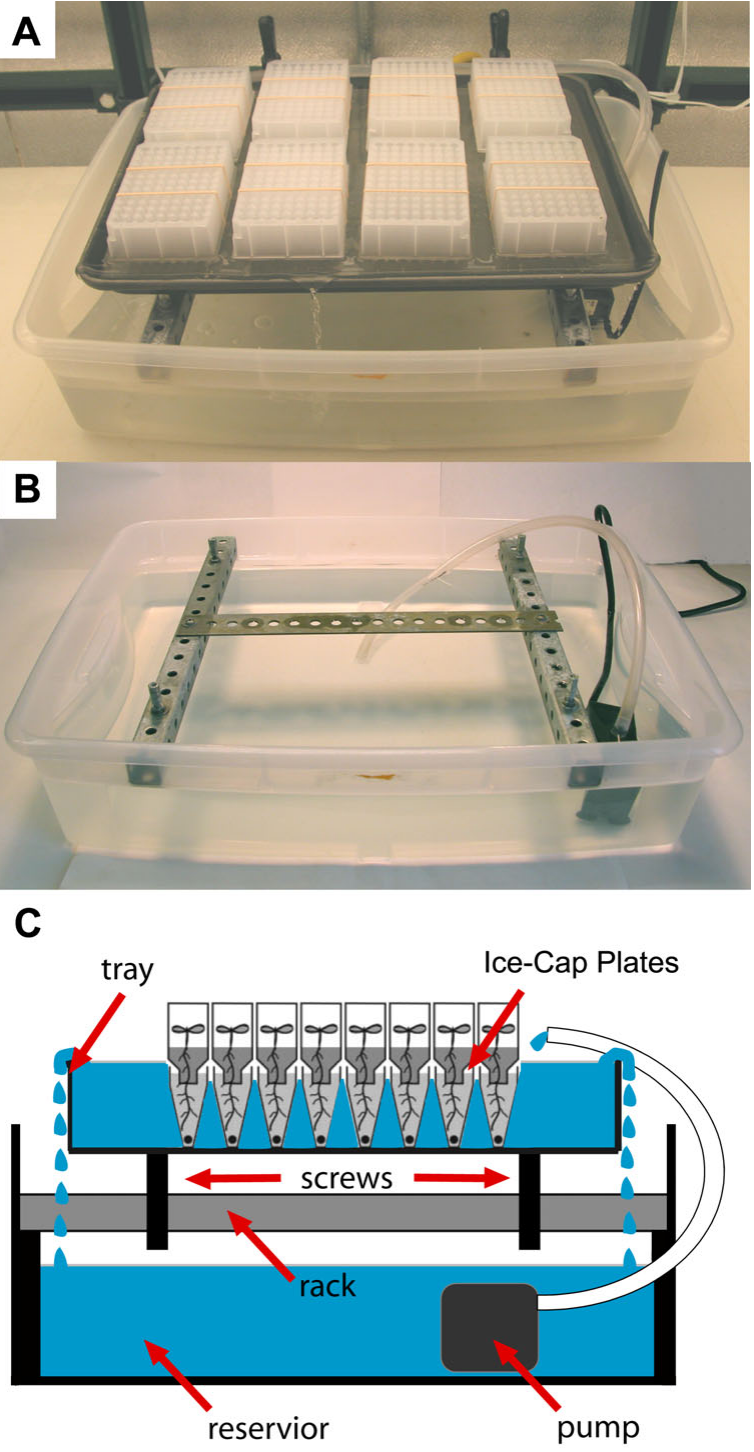
**Continuous watering system**. (A) Photograph of continuous watering system with 8 Ice-Cap plates in the upper tray. (B) Photograph of continuous watering system with Ice-Cap plates and metal tray removed to reveal metal rack. (C) Schematic of continuous watering system. Seedlings in Ice-Cap plates are placed in a rimmed metal pan. The pan is held in place over the larger reservoir by a metal rack. Screws in the rack allow the pan to be leveled. Water from a lower reservoir is constantly pumped into the pan and overflows back into the reservoir. The continuous flow of water into the upper pan ensures that the water level in the pan is always at the correct depth to keep the lower root plates of the Ice-Cap plates filled with water. Drawing is not to scale.

The key innovation in Ice-Cap 2.0 is a novel automatic watering system (Fig. 1). Briefly, seedlings in Ice-Cap plates are placed in a rimmed metal pan filled with water. The depth of the metal pan is carefully chosen so that when the pan is filled to capacity the water level in the pan is just deep enough to cover the tops of the wells of the lower root plates. Because the upper 96-well seedling growth plates do not form watertight seals with the lower root plates, water from the metal pan flows freely into the wells of the lower root plates, maintaining a constant water level in these wells. Depending on the particular metal pan that is chosen, it may be necessary to place a thin glass sheet on the bottom of the metal pan to raise the lower root plate up to the ideal depth with respect to the water level.

As described above, the addition of this type of metal tray alone does not solve the problem of keeping the lower plate constantly hydrated because the water level in this tray would rapidly drop over the course of a single day due to evaporation. To address this problem we suspended the pan above a larger reservoir filled with several liters of water. Adjustable screws allow the upper pan to be leveled to achieve uniform water depth. A submerged pump constantly pumps water from the high-capacity lower reservoir to the upper pan. The continual overflow from the upper pan spills back into the lower reservoir and ensures that the water level in the upper pan is constantly at the correct level to keep the lower root plates filled with water while avoiding submerging the seedlings. Water is added to the lower reservoir as needed so that the level does not drop below that of the recirculating pump, typically once per week. The system is large enough to accommodate eight Ice-Cap plates in a single fountain, allowing 768 seedlings to be grown in a 60 cm by 40 cm space.

## Protocol

In the original Ice-Cap system [1], *Arabidopsis thaliana *or rice seedlings are grown in a 96-well format by placing a single seed in each well of a 96-well upper seedling plate that contains nutrient agar. As the seedlings develop, the roots penetrate the agar and grow down through a hole in the bottom of the upper seeding plate into a lower 96-well plate containing water and a metal ball. The upper and lower plates are held together by elastic bands during the growth period. After ca. two weeks of growth the roots reach the bottom of the lower root plate. Tissue harvest is then performed by transferring the stacked plates to a dry ice/ethanol bath to flash freeze the water in the lower root plate. The resulting ice captures the root tissue, but does not freeze the seedlings in the upper plate. The upper and lower plates are then manually separated, yielding an upper plate containing 96 viable seedlings that can be transplanted to soil and a lower plate containing 96 root tissue samples. Genomic DNA is extracted from the root tissue by adding extraction buffer to the lower root plate, sealing it with foil, shaking it to pulverize the root tissue and centrifuging to pellet the cell debris. The resulting aqueous solution contains genomic DNA of suitable purity to be added directly to a PCR reaction for genotype analysis. When *Arabidopsis *seedlings are processed using this protocol the average yield of genomic DNA is 400 ng per sample, which is sufficient to perform hundreds of PCR reactions [1].

One shortcoming of the original Ice-Cap system was that seedling growth was sensitive to subtle changes in environmental conditions. The humidity level and light intensity of the growth chamber affected the performance of the original Ice-Cap system such that optimal growth could only be achieved within a narrow window of conditions. If the humidity was too low, the water level in the lower root plate dropped quickly and exposed the roots of the growing seedlings. If the water level dropped below the tip of the root, the seedling would die. Manually adding water to the lower root plate is not an option because the process of separating the upper and lower plates to add water would damage the roots and risk cross-contamination between adjacent wells.

To address this problem, we developed an automatic watering system that maintains a constant water level in the lower root plate for the duration of the growth period. Here we present a protocol detailing this novel watering system as well as other improvements to the Ice-Cap system.

### A. Constructing the Continuous Watering System

1. Place the small submersible pump in the bottom of the large plastic reservoir. Place the metal support rack in the reservoir container. Place the metal tray on top of rack and level by adjusting the levelling nuts on the support rack. Clamp the outflow hose from the pump onto the rim of the metal pan and fill the reservoir with water. **NOTE**: *We found that a mixture of one part tap water to three parts distilled water provides better growth performance than pure distilled water. The reservoir is typically filled with a total volume of 20 L of water. Subsequent re-filling of the reservoir to replenish water lost to evaporation should be performed using pure distilled water*.

2. Turn on the fountain pump and allow the metal tray to fill with water. Check the water level in the metal tray as follows. Fill each well in a 96-well lower root plate to capacity with water using a multichannel pipette. Place an empty 96-well upper seedling plate on top of the lower root plate and place the stacked plates in the water filled tray. Visually inspect the plates to make sure that the water level is just above the rims of the wells of the lower root plate. **NOTE**: *If the water level is too deep then it will be necessary to place a sheet of glass on the bottom of the metal tray. Choose a sheet of glass of a thickness that brings the lower root plate up to the desired depth relative to the water level*.

### B. Medium Preparation

We experimented with several different volumes of growth medium for the upper seedling plate and determined that seedlings grown in 525 μL of agar medium were larger, greener and grew more quickly than seedlings grown in the 125 μL of medium described in the original version of Ice-Cap (data not shown).

1. Prepare sterile agar growth medium composed of 0.5× Murashige and Skoog basal salt mixture, 2 mM MES, 0.6% agar, pH 5.7. Place the autoclaved medium in an incubator set at 65°C, which will prevent the medium from solidifying. (One liter of medium is sufficient for the preparation of 13 Ice-Cap seedling growth plates).

2. Perform the following operation in a sterile laminar flow hood. Seal the bottom holes of autoclave-sterilized 96-well upper seedling plates with removable adhesive sealing film using the hand roller. **NOTE**: *It is very important that the 96-well plates are completely dry and at room temperature before applying the sealing film, otherwise leakage will result when the agar medium is added in the next step*.

3. While the growth medium is still molten, use a multi-channel pipette to dispense 525 μL of growth medium into the bottom of each well. (Molten medium can be poured into disposable plastic troughs to facilitate pipetting). Leave the 96-well plates in the sterile hood while the medium solidifies. Once the medium has solidified and cooled to room temperature, cover the plates with a clear plastic lid, wrap in Al foil and store at 4°C for future use.

### B. Plating Seeds

1. Surface sterilize *Arabidopsis *seeds by soaking them in 95% (v/v) ethanol for 5 min, remove excess ethanol and allow them to air dry. **NOTE**: *It is very important that the seeds are free from any small particles of soil, plant tissue, or other debris that could fall into the holes in the seed loader*.

2. Use the 96-well seed-loading device to place one seed on the surface of the agar in each well. This is done by sprinkling the surface of the seed loader with several hundred seeds and using a soft bristle paintbrush to spread seeds evenly across the surface of the loader. A single seed should fall into each well in the seed loader. Use the paintbrush to carefully brush off excess seeds so that there is a single seed in each hole and no loose seeds on the surface of the seed loader. Place an upper seedling plate upside down on the seed loader and use the metal notches on the seed loading device to position the plate so that each well in the seedling growth plate is aligned over a single seed in the seed loader. Firmly hold the upper seedling plate against the seed loader and quickly invert the plate and loader to allow the seeds to fall into the wells in the upper seeding plate. Firmly tap the back of the metal plate to dislodge any seeds that may be stuck in the seed loader. Remove the seed loader from the top of the seedling growth plate. There should now be a single seed on the surface of the agar in each well of the upper seedling plate.

**NOTE**: *Seeds can also be plated individually. For example, we found that individual seeds can be transferred using a small (20–200 μL) sterile pipette tip attached to the end of a disposable 2 mL glass pipette (e.g. Fisher-Scientific 5 3/4" Pasteur Pipettes, catalogue no. 13-678-6A). The end of the pipette tip is first moistened by dabbing the agar in the upper seedling plate and pick up a single seed. The glass pipette is used as a handle to allow you to gently place a single seed on the surface of the agar in each well of the upper seedling plate*.

3. Place a clear plastic lid on each plate and seal the gap between the lid and the upper seedling plate with surgical tape.

4. Wrap the plates in foil and place them at 4°C for 2–7 d to stratify the seeds. This treatment improves germination rate and synchrony.

### C. Seedling Growth

**Figure 2:**
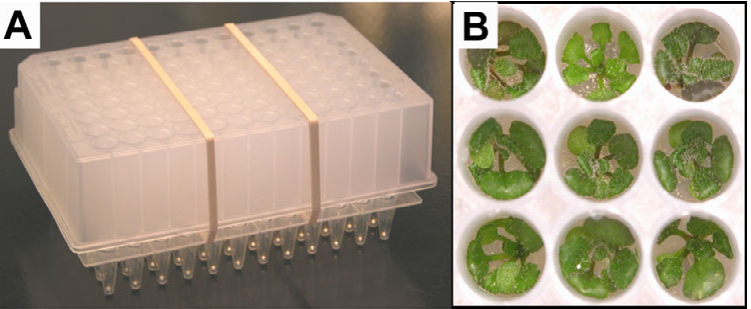
**Seedlings growing in Ice-Cap plates**. (A) Photograph showing a stacked upper seedling plate and lower root plate ready for transfer to the continuous watering system. (B) Seedlings grown for 15 d at 21°C in continuous light using Ice-Cap 2.0.

In the original Ice-Cap method the clear plastic lids were left on the upper seedling plates for the duration of the growth period. We have now determined that removing the clear plastic lids from the upper seedling plates after 2 d of growth and leaving them off for the remainder of the growth period allows for much more vigorous seedling growth (Fig. 2). Removing the lid helps dissipate heat from the plates, which allows the Ice-Cap system to perform better under higher light intensities. We originally expected that growing the seedlings with no lid would lead to substantial bacterial or fungal contamination of the agar medium. In practice we have found that contamination is very rare. Even when contamination was detected, it did not affect plant survival.

**Figure 3:**
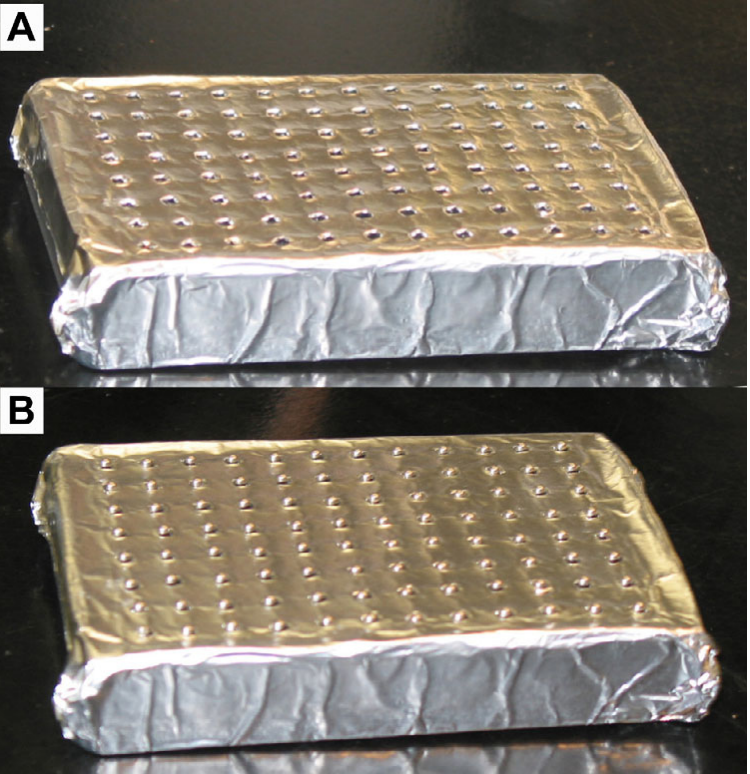
**Metal ball dispenser**. (A) Photograph of the home-made 96-well metal ball dispenser without metal balls. (B) The same device with 96 metal balls loaded on top of it. The ball-loading device was created by placing several sheets of Al foil over the surface of a 96-well PCR plate and carefully marking the location of the centre of each well on the foil using a marking pen. The foil was transferred to the plastic lid from an empty pipette tip box to provide structural support. Each pen mark was carefully widened using a small stick to create a divot just large enough to hold one of the 3/32" diameter metal ball bearings that are placed in the bottom of the root growth plates.

1. Remove the foil from the upper seedling plates following their incubation at 4°C and transfer them to 21°C under constant fluorescent light. At this stage the growth plates still have the plastic sealing film on the bottom and clear plastic lid on the top and are not stacked on top of a lower root plate.

2. After 2–3 d incubation under constant light, remove the lids from the upper seedling plates and place the upper seedling plates on top of lower root plates as follows. To begin with, prepare the lower root plates by placing a single metal ball bearing into each well of the lower root plates. A simple home-made device can be used to dispense single metal balls into the lower root plates (Fig. 3). Place the ball-loading device in a large dish to catch excess metal balls. Sprinkle metal balls over the surface of the ball loading device and gently shake to allow a single ball to rest in each divot of the ball loading device. Place a lower root plate upside down on the ball loading device and hold firmly together. Invert the plate and ball loader so that the metal balls fall into the wells of the lower root plate. Use a multi-channel pipette to fill each well of the lower root plate to capacity with a mixture of distilled water/tap water at a ratio of 3 parts distilled water to 1 part tap water. Carefully peel the sealing film from the bottom of the upper seedling plate. Place the upper seedling plate on top of the lower root plate and secure the plates together with elastic bands. **NOTE**: *Be sure to align the upper seedling plate and the lower root plate so that the nibs on the bottom of the upper seedling plate fit securely in the raised rim of the lower root plate. Also make sure that the plates are in the same orientation (i.e. the nib from well A1 in the upper seedling plate is placed in well A1 of the lower root plate*).

3. Place the stacked plates in the continuous watering system and grow under constant light until root tissue is easily visible in the bottom of the lower root plate. This typically occurs 11–13 d after the plates have been transferred to the continuous watering system.

### D. Tissue Collection and DNA Extraction

**Figure 4:**
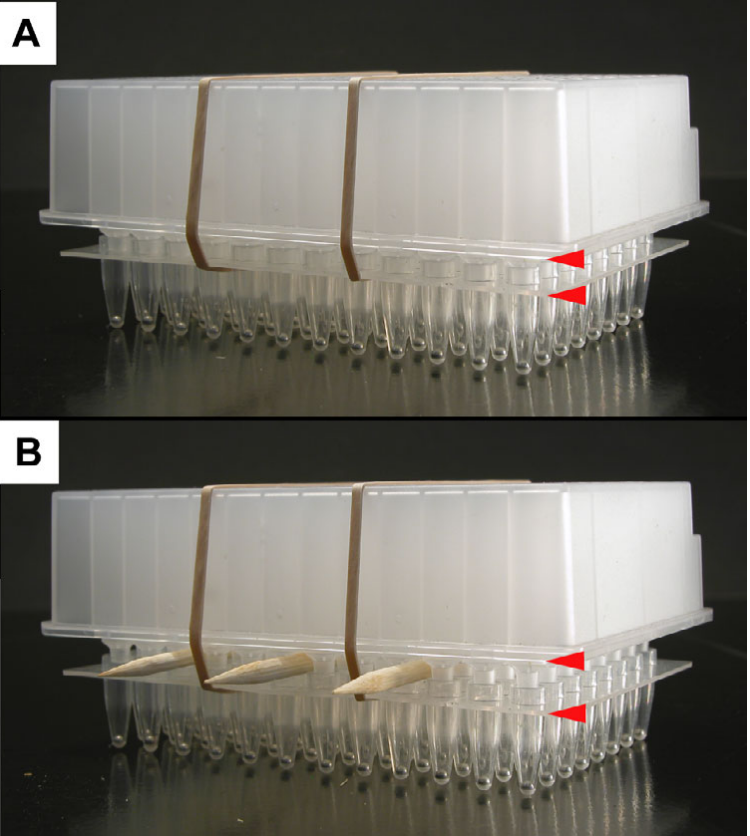
**Ice-Cap plates with wooden skewers**. (A) Stacked Ice-Cap plates prior to skewer insertion. (B) Stacked Ice-Cap plates with wooden skewers inserted between upper and lower plates to expedite the drop in water level in the wells of the lower root plate before freezing for tissue harvest. Red arrowheads indicate the distance between the upper and lower plates.

Before tissue harvest, it is necessary to allow the water in the lower root plate to drop below the level of the nibs of the upper seedling plate. In the original Ice-Cap, this drop in the water level occurred as the seedlings grew and depleted their water supply. With the continuous watering system, the lower root plate is constantly filled with water. For this reason the stacked Ice-Cap plates are removed from the continuous watering system 1–2 d before tissue harvest. The plates are incubated in the same growth room at 21°C under constant light to allow the water level in the lower root plates to drop through evaporation and transpiration. Wooden skewers are inserted between the upper and lower plates at this time to expedite the drop in water level (Fig. 4). The skewers also make it easier to separate the upper and lower plates after freezing the lower root plate for tissue harvest.

1. Remove the stacked Ice-Cap plates from the continuous watering system and set them on a shelf in the growth chamber at 21°C under constant light. Leaving the elastic bands in place, carefully insert wooden skewers between the upper and lower plates to create an air space between the two plates and hasten the drying process (Fig. 4). It typically takes 1–2 d for the water level to drop below the level of the nibs of the upper seedling plate.

2. Chill the 96-well metal thermal block in a dry ice and 95% (v/v) ethanol mixture. Place the metal thermal block in freeze-resistant glass dish. Place dry ice in dish around the metal thermal block. For a 2-L (28 cm × 18 cm × 4 cm) dish we use about 1 kg dry ice. Carefully pour 95% (v/v) ethanol into the glass dish until the level of the dry ice and ethanol is just below the surface of the metal thermal block. Allow the metal thermal block to chill for about 20 min to equilibrate.

3. Place the wells of the lower 96-well root plate into the holes in the thermal block with the upper seedling plate still held in place by the elastic bands and the skewers still in place between the plates. Leave the stacked plates on the thermal block for 5 min, during which time the water in the lower root plate will freeze solid. Take the stacked plates from the thermal block, remove the elastic bands, and remove the skewers from between the plates. Carefully separate the upper and lower plates. **NOTE**: *The best way to separate the two plates is to peel the upper seedling plate away from the lower root plate in the same manner you would peel the backing off a sheet of adhesive film. During this process the roots that are frozen in the lower root plate break off at the level of the ice, leaving a sample of frozen root tissue in the lower plate and a viable seedling in the upper plate. Leaving the skewers in place during the freezing of the lower root plate facilitates the separation of the upper and lower plates after the water in the lower root plate had frozen solid*. **NOTE**: *Following tissue harvest, the upper seedling plates should be transferred to fresh lower root plates filled with water. The stacked plates can then be returned to the continuous watering system to allow the seedlings to continue growing (see below)*.

4. Allow the water in the lower root plates to thaw at room temperature. Add 25 μL of Tris-EDTA buffer (500 mM Tris pH 8, 50 mM EDTA pH 8) to each well. Seal the lower root plates using thermal adhesion foil and a manual heat sealer.

5. Load the sealed plates containing water, root tissue, Tris-EDTA buffer and the metal ball bearings into the Genogrinder machine. Shake for 5 min at 1,500 strokes/min. Remove plates from the Genogrinder and spin them in a centrifuge equipped with microplate carriers for 10 min at 3,500 rpm at room temperature to pellet cell debris. Carefully peel off the sealing foil and transfer 20 μL of the supernatant to a clean 96-well plate containing 180 μL of distilled water. This diluted sample can be used as template for PCR genotyping reactions and any unused samples can be placed at -20°C for long-term storage.

### E. Transplanting Selected Seedlings for Seed Production

Following tissue harvest, the upper seedling growth plates should be placed in fresh lower root plates and returned to the continuous water system. The seedlings can be maintained in these 96-well seedling growth plates for up to two additional weeks of growth. During this time period genotype analysis can be performed on the DNA samples collected from each plate of seedlings. When individual plants of the desired genotype are identified, those seedlings can be easily transferred out of the 96-well growth plate and into soil using the procedure described below.

1. Remove the lower root plate from the upper seedling plate. Any root tissue that has grown down into the new lower root plate must be snipped off to allow the seedling to be easily removed from the upper seedling plate. This second removal of root tissue does not affect seedling survival (data not shown).

2. Two different approaches can be used to remove individual seedlings from the seedling growth plate:

Option 1. Place a rubber hose over the opening in the bottom of the well containing the seedling of interest and connect the other end of the hose to a compressed air source. Carefully increase the air pressure until the plug of agar containing the seedling slides out of the well onto the lab bench. **NOTE**: *Care must be taken when performing this operation so that the seedling of interest does not inadvertently shoot across the lab. It is advisable to practice this technique using control seedlings before attempting to collect rare individuals with a desired genotype*. Transfer the entire agar plug containing the seedling to soil, making no effort to remove the agar from the root system. The agar plug containing roots is buried in the moist soil, leaving the hypocotyl and leaves exposed to the air.

Option 2. Use a wooden skewer to carefully push the agar plug out the top of the upper seedling plate. **NOTE**: *The blunt end of the skewer usually works better for this than the sharp end. It is also possible to use a combination of the pressurized air and wooden skewer approaches to remove desired seedlings from the seedling growth plates*.

3. Cover the transplanted seedlings with a plastic dome to maintain high humidity for the first 2 d following transfer to soil.

## Comments

The continuous watering system described in this paper provides an effective solution to the problem of sub-optimal seedling growth that can be encountered when using the originally described Ice-Cap system. The continuous watering system and other modifications to the Ice-Cap method have improved the robustness and utility of the system. The optimized protocol provides a cheap, simple and effective method for extracting genomic DNA samples from thousands of viable plant seedlings. We are currently using the Ice-Cap system to analyze the progeny of *Arabidopsis thaliana *plants that are segregating T-DNA insertions at multiple loci. This application is one of several possible uses for the Ice-Cap system, which include map-based cloning and screening for rare recombinants between tightly linked mutant loci. We have previously shown that the Ice-Cap method can be used with rice seedlings [1] and it is expected that the method could also be applied to a wide variety of other plant species.

## Competing interests

The author(s) declare that they have no competing interests.

## Authors' contributions

KAC participated in the design of the study, tested the continuous watering system and other improvements and drafted the manuscript. PJK conceived of the study and participated in its design and coordination and helped to draft the manuscript. All authors read and approved the final manuscript.
